# Fascia Lata and Tensor Fasciae Latae in Lower-Limb Reconstruction

**DOI:** 10.3390/jcm15145547

**Published:** 2026-07-15

**Authors:** Nina Szczerba, Jagoda Waleszczyńska, Dominika Jerka, Klaudia Bonowicz-Kozłowska, Maciej Gagat

**Affiliations:** 1 Histological Research Club, Department of Morphological and Physiological Sciences, Faculty of Medicine, Collegium Medicum, The Mazovian University in Płock, 09-402 Płock, Poland; ninkasz5@wp.pl (N.S.); jagoda.waleszczynska@gmail.com (J.W.); 2 Department of Histology and Embryology, Collegium Medicum in Bydgoszcz, Nicolaus Copernicus University in Torun, 85-092 Bydgoszcz, Poland; dominika.jerka@cm.umk.pl (D.J.); klaudia.bonowicz@cm.umk.pl (K.B.-K.); 3 Department of Morphological and Physiological Sciences, Faculty of Medicine, Collegium Medicum, The Mazovian University in Płock, 09-402 Płock, Poland

**Keywords:** fascia lata, reconstructive surgery, tensor fasciae latae, vascularized fascial graft

## Abstract

The fascia lata (FL), the deep fascia of the thigh, plays a crucial role in lower-limb biomechanics, force transmission, and surgical applications. This is because of its histological architecture and, more importantly, its integration with the major lower limb muscles. Its regionally variable thickness and multilayered organization enable the formation of extensive attachments to the iliac crest and deep crural fascia, functionally integrating it with the tensor fasciae latae, gluteus maximus, and iliotibial tract. Once a passive envelope surrounding the thigh musculature is considered, it is now understood to be a dynamic collagen-rich structure with both mechanical and biological roles. Understanding the vascularization and innervation patterns of the fascia lata is crucial for both surgical procedures and regenerative medicine, as these features influence the healing potential and graft viability. Close anatomical and functional integration between the FL and tensor fasciae latae (TFL) enhances the biomechanical strength and vascular potential of FL-based grafts, effectively resulting in the structure being a promising resource for advanced reconstructive surgery. This relationship provides not only mechanical reinforcement, but also improved biological viability, making vascularized FL particularly valuable in situations that require durable tissue support. Emerging evidence highlights its potential in managing avascular bone necrosis, where TFL contributes to revascularization and enhances the healing capacity. Although FL has been applied in select lower-extremity reconstructions, its broader reconstructive possibilities remain underexplored. The combination of structural resilience, biological activity, and adaptability underscores FL and TFL as underutilized yet highly promising grafts for modern orthopedic reconstruction.

## 1. Introduction

“Fascia” is a word derived from Latin meaning bandage. Historically applied to a range of undifferentiated mesenchymal tissues, the term is now recognized to denote structures with an important role in the biomechanics of the human body [1,2,3]. Epimuscular force transmission is the force transferred from one muscle to another or to nearby structures [4]. Therefore, the fibers received by the FL from the gluteus maximus (GMAX) and TFL are thought to contribute to force redistribution across the lateral aspect of the thigh [5]. These examples highlight the necessity for a detailed understanding of both anatomy and histology to appreciate FL functions and any changes that may occur secondary to dysfunction or pathology. Therefore, a comprehensive narrative review of the literature was undertaken to assess morphological and clinical information, such as attachment sites, correlation with certain muscles of the lower limb, innervation patterns, and clinical potential. The novelty of this review lies in integrating, within a single reconstructive perspective, the anatomical, vascular, histological and biomechanical characteristics of the fascia lata and tensor fasciae latae, structures that are most often considered separately. On this basis, the review is framed around the premise that the close anatomical and functional integration of the FL and TFL underlies their value as vascularized grafts, and that their combined potential in lower-limb salvage and in the management of avascular bone necrosis remains underexplored relative to more established reconstructive options.

## 2. Anatomy

**Figure 1:**
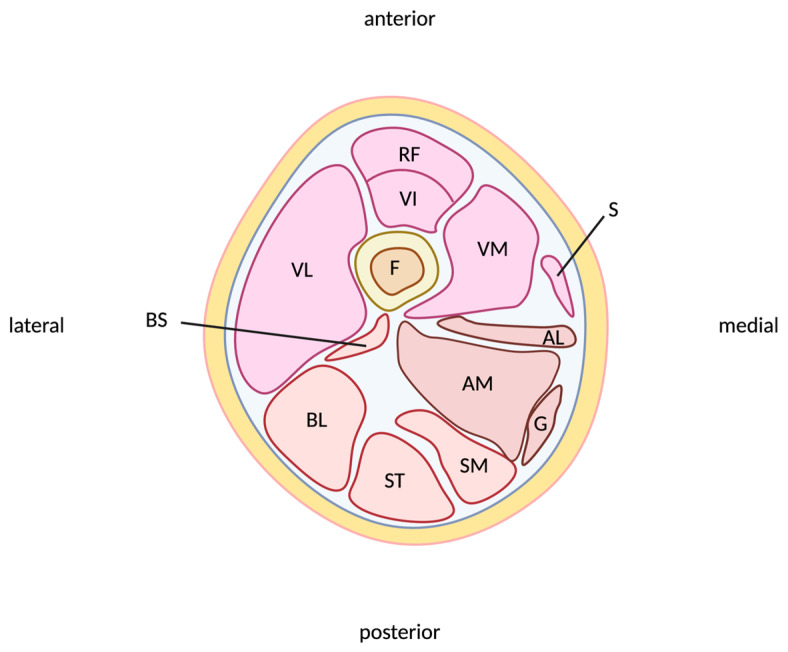
Cross-section of the thigh at mid-thigh level. Abbreviations: F, femur; RF, rectus femoris; VI, vastus intermedius; VL, vastus lateralis; VM, vastus medialis; S, sartorius; Al, adductor longus; AM, adductor magnus; G, gracilis; SM, semimembranosus; ST, semitendinosus; BL, long head of biceps femoris; BS, short head of biceps femoris. The color pink indicates the anterior compartment, orange indicates the posterior compartment, and brown indicates the medial compartment.

The literature distinguishes between superficial and deep fasciae, although in clinical practice, the term “fascia” often refers to the deep fascia [1]. The FL gives rise to medial, lateral, and posterior femoral intermuscular septa that extend all the way to the shaft of the femur, dividing the thigh region into three osteofascial compartments: the anterior, medial, and posterior compartments [2] (Figure 1). The walls of these three compartments branch from the deep fascia and attach to linea aspera. The lateral intermuscular septum is particularly strong, while the other two are relatively weaker [3,4].

### 2.1. Anatomical Complexity

**Figure 2:**
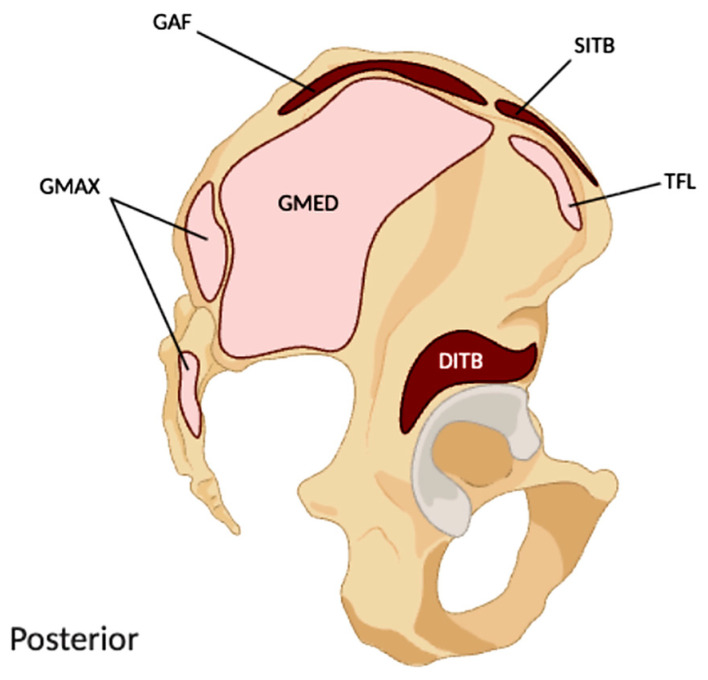
The FLAIC’s anatomy. Abbreviations: GMAX, gluteus maximus; GMED, gluteus medius; GAF, gluteal aponeurotic fascia; SITB, superficial layer iliotibial band; DITB, deep layer iliotibial band; TFL, tensor fascia lata.

A prime example of the structural complexity of the deep fascia is observed in the FL. This dense fibrous layer envelops the musculature of the thigh and extends superiorly to the gluteal and iliac regions. Notably, the TFL muscle inserts into the FL [6] and GMAX, which is the largest skeletal muscle in the human body [7]. The FL is thickened on the superolateral thigh [7], inferior to the TFL muscle attachment at the anterior lip of the iliac crest [8]. Proximally, it splits, encloses the TFL muscle, and anchors this muscle to the iliac crest [9] as the fascia lata attachment at the iliac crest (FLAIC) (Figure 2). It continues longitudinally as the iliotibial band (ITB) through the lateral thigh [7]. As it approaches the knee, the ITB separates into two functional components: the iliopatellar band and iliotibial tract (ITT). The first component attaches distally to the lateral condyle of the tibia (Gerdy’s tubercle), whereas the second component is at the lateral patellar retinaculum [10].

Anteriorly, the iliopatellar’s fibers attach slightly posterior to the Gerdy tubercle and posteriorly anterior to the fibular head [11]. GMAX has two distinct portions, where the superior portion inserts into the ITB and the inferior portion inserts into the femur at the linea aspera [12]. The gluteal aponeurotic fascia, which overlays the gluteus medius, comprises the posterior element of the FL complex and anchors it to the posterior outer lip of the iliac crest. Its fibers also integrate with the ITB, reinforcing the lateral fascial tension system of the thigh [13]. Distally, the FL continues as the crural fascia, attaching to the femoral and tibial condyles and head of the fibula [2].

Specialized thickenings of the deep fascia, known as the retinacula, occur near joints such as the knee, where they stabilize tendinous structures and provide a low-friction interface for tendon gliding [14]. Distally, some fibers of the FL and ITB, mostly the ITT, join the lateral and medial edges of the patellar ligament, forming the retinacula patellae [2,15]. The ITB provides an oblique myofascial expansion that passes over the patella, contributing to the formation of the anterior knee retinaculum and stretching the crural fascia in a lateromedial direction [16]. In the upper FL, an ovoid opening known as the saphenous hiatus, located below the inguinal ligament, is closed by the cribriform fascia and transmits the saphenous vein and associated lymphatic vessels [2,3].

### 2.2. Vascularization

The FL, as a deep fascial compartment, is notably vascularized, although almost no distinct data exists on whether the FL has its own reliable vascular supply. Most studies show that the FL receives its own vascular supply from the perforators of the descending branch (DB) originating from the lateral circumflex femoral artery (LCFA) [17]. The TFL muscle is mainly vascularized by the ascending branch (AB) of the LCFA [18], which traverses the TFL muscle usually 6–10 cm distal from the anterior superior iliac spine [19]. However, recent studies have shown that some TFL muscles are not exclusively supplied by the AB of the LCFA. Some of them receive dual blood supply from both the AB and transverse branches (TB) of the LCFA, dividing into two to four more branches before entering the muscle [20]. In addition, the superior gluteal artery, by its deep branch, contributes a secondary vascular supply to the TFL muscle [19]. The proximal end of the ITB is vascularized by the AB and TB of the LCFA, as well as the superior gluteal artery [19], but the distal end of the ITB is avascular [21].

### 2.3. Innervation

The lumbosacral plexus gives rise to several nerves that contribute to FL innervation. Among these are the ilioingual nerve (L1), femoral branch of the genitofemoral nerve (L1–L2), lateral femoral cutaneous nerve (L2–L3) and cutaneous branch of the obturator nerve (L2–L4). These nerves are primarily cutaneous and innervate specific regions of the skin; therefore, to reach the superficial layers, they must perforate the FL, thereby providing sensory innervation [22]. The TFL muscle is innervated primarily by the superior gluteal nerve (SGN) (L4, L5, and S1 roots) [19,23]. Usually, the intramuscular branch of the SGN enters the muscle as a single branch 6.87 ± 1.26 cm from the anterior superior iliac spine, in the proximal 10–25% of the TFL muscle’s length, dividing into two branches inside, traveling forward and downward, following a deeper course as they extended further inferiorly and distally [24]. The ITB shares the same innervation with the TFL muscle, with minor inferior gluteal nerve influence [19,25].

## 3. Histology

Fasciae are extensive connective tissues that envelope muscles, nerves, organs and viscera [26].

### 3.1. Cellular Elements

The FL is a dense regular connective tissue made of tightly aligned fibers, with sparse elongated fibroblasts embedded and orientated along the femoral axis [27]. It is mainly composed of type I collagen fibers that run parallel, predominantly in the longitudinal direction, and sometimes in the crossing direction with much less presence of elastin [28]. It is also rich in periosteal mesenchymal stem cells confined to the cambial layer [29]. Between the ITB and the lateral aspect of the femur, adipose tissue is highly vascularized and richly innervated and sometimes contains Pacinian corpuscles. Fibrous strands anchor the ITB to the femur, the cause of which is divided into proximal and distal ligamentous parts [9].

### 3.2. Tissue Organization

The superficial fascia consists of a fibrotic layer separating the superficial from the deep adipose tissue and allowing movement between the skin and deeper structures [1,30]. Microscopically, it appears as a multilayered structure with a mean thickness of 146.6 ± 31.5 µm, thicker proximally and thinning towards the knee, where it almost adheres to the deep fascia [31,32]. The deep fascia is a three-dimensional continuum of dense, fibrous connective tissue organized in sheet-like structures, encasing bones, muscles, tendons, nerves, and blood vessels [1,33]. From a microscopic viewpoint, it appears as a multilayered structure formed by two or three layers of collagen fibers separated by loose connective tissue [31]. This tissue is exposed prior to the reflection of the ITB and biceps femoralis, located between these two superficial structures distally toward the tibia [11]. While the deep fascia is connected more loosely to the skin, it participates in extensive anchoring through cutaneous ligaments, which are collagenous structures that span from the deep fascia to the dermis. These ligaments play a key role in maintaining integumentary stability against mechanical forces such as gravitational stress [1]. Strong mechanical properties are associated with high collagen content [29].

### 3.3. Telocytes

Telocytes (TC) are a relatively recent discovery, first identified in 2005, within the stromal tissues of various organs. Initially, because of their resemblance to the classic interstitial cells of Cajal in the gastrointestinal tract, they were referred to as Cajal-like cells. Since 2010, after confirming their widespread presence and detailed characterization through immunohistochemistry and electron microscopy, this new type of cell has been known as a telocyte. TCs are cells with a small body and various prolongation names called telopodes [34,35]. In the FL, TCs are specialized connective tissue cells sensitive to mechanical signals that are capable of coordinating or assisting with other cells, such as stem cells, fibroblasts, and macrophages, in various processes linked to tissue repair, regeneration, and structural adaptation throughout the organism [35,36].

Considering that TCs are quite a new feature, their functions have not yet been scientifically proven. They may serve as a form of mechanical support owing to their three-dimensional network structure, which is both resilient and adaptable. TCs may influence vascular dynamics, such as vessel closure or blood flow. Additionally, their presence in neuromuscular spindles may be linked to the control of muscle tension and motor activity [34,37].

### 3.4. Microstructure

The overall elemental concentration of FL is mostly influenced by the amino acid composition of collagen, that is, glycine (33.5%), proline (12%), and hydroxyproline (10%) [38]. In addition to the highest content of ubiquitous macroelements, such as carbon (C), oxygen (O), and nitrogen (N), with median values of 40.78, 33.69, and 19.03%, (23173122) phosphorus (P) and calcium (Ca) showed high concentrations, ranging from 216 to 349 and 233 to 367 µg∙g^−1^, respectively. Elevated levels are also noted for magnesium (Mg) and zinc (Zn), with concentrations between 44.9 and 48.1 µg∙g^−1^ for Mg and between 9.66 and 13.4 µg∙g^−1^ for Zn. Lower amounts were recorded for aluminum (Al), copper (Cu) and iron (Fe), ranging from 3.33 to 6.51, 0.98 to 1.57 and 5.68 to 7.84 µg∙g^−1^, respectively. Boran (B) and strontium are present in smaller quantities, measured between 0.5 and 1.0 µg∙g^−1^. Trace levels (less than 0.4 µg∙g^−1^) are found for silver (Ag), barium (Ba), cadmium (Cd), chromium (Cr), manganese (Mn), nickel (Ni), lead (Pb) and titanium (Ti) [39]. Female-derived fascia lata has increased levels of Ba, Ca, Cr, Sr, and Zn compared to male-derived fascia, which shows higher concentrations of Fe, P, and Mg [39]. Aging is associated with a reduction in Al, Ca, and P levels in the human fascia lata, yet a distinct rise in Ca is observed noticeably over time [39].

## 4. Biomechanics

This complex system, which includes the FL, TFL, ITB, GMAX, and retinacula, forms an integrated system that connects the pelvis, hip, and knee. Their biomechanical role extends far beyond passive support (Table 1).

### 4.1. The FL

The fascial tissue system has two main functions, mechanical and protective. These are mainly ensured by the deep fasciae. It allows load transfer and provides stability and joint connections while wrapping individual tissue structures, maintaining their shapes and positions [40]. The FL, TFL, and GMAX play an important role in monopodal balance, allowing the knee to swing along the hip. This complex structure is known as the ‘pelvic deltoid’ [41].

### 4.2. The TFL

The main mechanical function of the TFL muscle is hip abduction [12]. However, it also assists the rectus femoris in hip flexion [42] and internal hip rotation [43]. The role of the knee joint has been widely discussed, although new studies have shown that it is an accessory knee flexor once the knee is flexed beyond 30°, and then works with the ITB to stabilize the joint in full extension [42]. The TFL plays a particularly important role in walking. The muscle was significantly more active during the swing phase by pulling the ilium down on the weight-bearing side, which caused the other hip to rise. Thus, the non-weight-bearing leg avoids hitting the ground during the swing phase [44].

### 4.3. The ITB

The ITB is an important structure for the biomechanics of both hip and knee joints.

At its proximal end, the ITB plays a role in maintaining lateral hip stability by countering inward movement of the thigh [45]. As adduction increases, tension within the ITB tends to increase [45]. Owing to its attachments to the pelvis, femur, and tibia, the ITB is responsible for restraining the tibial internal rotation and pivot shift phenomenon as the knee flexes [46,47,48]. It is also a component of the reserve extensor apparatus of the knee joint, taking part in the FL in lateral stabilization, securing it against bending. As a knee’s anterior side stabilizer, it functions as a synergist of knee flexion and extension, contributing to the rotational movements of this joint [41]. Both the FL and ITB participate in leading the patella [48]. The ITB attachments to the patella stabilize it against medial dislocation [12]. Two fiber bundles of the ITB, called Kaplan fibers, were identified. The proximal one courses from the undersurface of the superficial ITB to the distal femur in an almost transverse orientation, 53.6 mm proximal to the lateral epicondyle, while the distal one courses from the superficial ITB and transverses from proximal and lateral to distal and medial before insertion to the femur, 31.4 mm proximal to the lateral epicondyle [48]. Both Kaplan fibers provide rotatory stability to the knee. They, especially the distal fiber bundle, play a key role in the anterolateral aspect of knee stability. They tighten during internal rotation of the tibia at 30° flexion [49].

### 4.4. The GMAX

The GMAX, with its fascial insertion into the ITB, plays a major role in maintaining the lateral stability of the pelvis during walking, rather than abduction and/or flexion [6,47]. However, GMAX is a primary hip extensor [50] and lateral hip rotator [51]. The superior portion of the GMAX via its attachment to the ITB assists in stabilizing the sacroiliac joint and transmitting loads from the limbs to the trunk [52].

### 4.5. The Retinacula

The retinacula are not just static structures for joint stabilization, but they also take part in the spatial proprioception of knee movements [14]. Instability of the patellofemoral joint is most likely during shallow flexion (0–30°), as the patella is not securely locked into the trochlear groove. The retinacula helps reduce this risk by directing the patella along its proper track [53]. The medial retinaculum plays an important role as a lateral stabilizing factor for the patella, especially during the early stages of knee flexion [54,55]. The lateral retinaculum helps control lateral patellar motion. Even a limited release of the lateral retinaculum leads to markedly greater lateral patellar displacement than a knee with preserved lateral stabilizing structures, with the most pronounced effect occurring in full flexion. This suggests that the lateral retinaculum may function as a significant restraint for lateral translation [56].

## 5. Clinical Potential of FL Using ALT (Anterolateral Thigh Flap)

Due to its vascular course and the surrounding structures, such as the TFL muscle and the overlying skin, FL is an excellent material widely used in various surgical and reconstructive procedures. Its robust architecture, reinforced with collagen fibers, makes it suitable for applications such as Achilles tendon reconstruction and ligament repair [57]. In many cases, FL is employed to augment grafts, providing adequate vascularization after the restoration of specific structures, enhancing stability, and improving overall tissue integrity.

### 5.1. Achilles Tendon Reconstruction with FL

Achilles tendon rupture is a relatively frequent injury, and operative treatment generally yields better functional outcomes than nonoperative care. Surgical repair is associated with a faster resumption of activity, improved restoration of strength and endurance, and a reduced likelihood of re-rupture. Despite these advantages, however, postoperative complications are common. Wound breakdown, infection, tendon exposure, and soft tissue necrosis may develop, largely because the distal third of the leg has a limited blood supply and contains thin, relatively immobile soft tissue that provides inadequate coverage [58,59]. In the context of the Achilles tendon, the literature describes a broad spectrum of associated pathologies range from ruptures and mechanical injuries to soft-tissue defects [60]. For Achilles tendon reconstruction, FL alone may be used; however, the most effective results are typically achieved with grafts that incorporate both muscle tissue and the fascia vascular network. Many of these procedures were initially performed in animal models because of their complexity, allowing researchers to verify their safety and expected clinical outcomes before their use in human patients [61].

### 5.2. ATL Flap with FL in Soft-Tissue and Tendon Reconstruction

Consequently, the free flap with FL has been proposed as the best option for combined defects because it allows for simultaneous vascularized reconstruction of the Achilles tendon and overlying soft tissue. Various types of free flaps have been proposed, including muscle, musculocutaneous, fasciocutaneous, and perforator flaps [62,63]. The extent of Achilles tendon involvement ranged from complete full-thickness defects to partial-thickness defects, encompassing more than half of the tendon. These injuries typically present with overlying soft-tissue damage accompanied by overlying soft-tissue loss [64].

Composite free ALT flaps incorporating FL have been widely applied in the reconstruction of complex Achilles tendon defects [65]. During ALT flap elevation, the FL was obtained from the more lateral region of the thigh. This approach provides reliable soft tissue coverage and a well-vascularized fascial component suitable for tendon restoration. The FL is then tubularized to function as a tendon substitute, effectively replacing the segmental Achilles tendon defect, while maintaining adequate vascularity through its connection to the ALT flap [66]. In some cases, the vastus lateralis (VL) fascia is used as an alternative to FL. Owing to its comparatively reduced thickness and mechanical strength, VL fascia requires additional reinforcement. Consequently, it is folded, either doubled or tripled according to the size and severity of the Achilles tendon defect, to provide sufficient tensile integrity for reconstruction [67].

### 5.3. Operative Procedure for Achilles Tendon Reconstruction with FL

Due to the complexity of the procedure, it may be performed by two surgical teams; however, this is not always feasible, as it depends on the surgeons’ working preferences and availability of medical personnel.

#### 5.3.1. Surgical Team 1: Flap Design and Harvest

**Figure 3:**
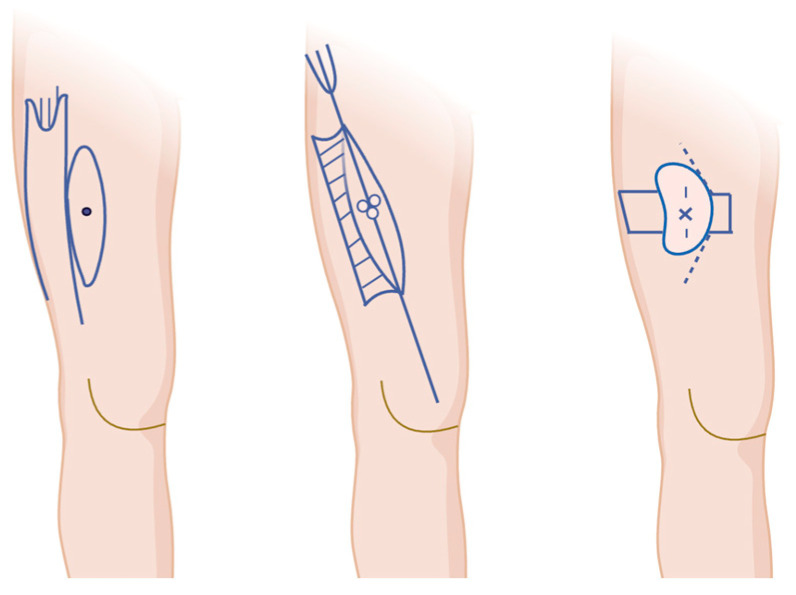
Schematic examples of fascia lata/TFL-based flap designs [68,70,71].

The patients were placed in the supine position. A line connecting the anterior superior iliac spine to the superolateral patellar border was divided into segments, and a 2.5 cm radius circle centered at the midpoint was used as the perforator search area. For surgeon convenience, the site designated for harvesting the FL during ALT flap preparation was marked in advance. However, the marking method varies depending on the operating surgeon and the individual preferences (Figure 3). Handheld Doppler mapping identified cutaneous perforators, after which an appropriately sized skin paddle was outlined [68]. An incision along the medial margin of the planned flap allowed elevation in the subfascial plane until the descending branch of the lateral circumflex femoral artery (LCFA) and its perforators were exposed. Two perforators arising from the descending branch were selected to create a chimeric construct consisting of an anterolateral thigh (ALT) skin paddle and separate FL flap [64]. Each component was harvested using its own perforators. When necessary, the skin paddle is microscopically thinned by removing the deep subcutaneous fat while preserving the subdermal vasculature. The FL component was approximately 2–5 cm longer than the Achilles tendon defect to facilitate tendon reconstruction. Both flaps were elevated and divided from the descending LCFA pedicle [68,69,70,71].

#### 5.3.2. Surgical Team 2: Recipient-Site Preparation and Reconstruction

Concurrently, necrotic tissue at the defect site was excised. The posterior tibial artery and venae comitantes were dissected for microvascular anastomosis. Either end-to-end or side-to-end anastomosis was performed before flap inset. Once adequate perfusion is confirmed, reconstruction is performed [72].

The FL flap was folded two–three times to create a tendon-like segment and secured to the residual Achilles tendon with 2-0 polypropylene interrupted sutures or anchored to the calcaneus using screws when necessary. The thinned ALT skin paddle was then positioned to resurface the cutaneous defect over a silicone drain [73]. All donor sites were primarily closed. Postoperatively, the ankle was immobilized in a neutral position with an above-knee splint for three weeks. Surgical management is indicated in patients presenting with a variety of conditions ranging from Achilles tendon ruptures to areas of tissue necrosis necessitating debridement prior to reconstruction. In such cases, the FL may be reconstructed using a direct graft harvested from the TFL and ITB [71].

### 5.4. Achilles Tendon Reconstruction ATL with FL in Pediatric Surgery the Procedure Differs in Pediatric Patients

In contrast to adults, it is essential to confirm the presence of the posterior tibial artery using Doppler imaging beforehand. The perforating branch along the planned flap axis was identified and the superior medial malleolus was marked. Following wound debridement, with the ankle held in 20° plantar flexion and the knee flexed to 30°, the extent of the Achilles tendon defect and the overlying skin loss were measured [74].

The procedure is performed in two stages:

The first stage involves reconstruction of the Achilles tendon using the FL [75]. A strip of FL, approximately 4–5 cm wide and 3 cm longer than the tendon defect was harvested from the ipsilateral thigh. The smooth surface was oriented outward and the graft was tubularized with atraumatic sutures. It was then anchored to the proximal and distal ends of the tendon. With the knee flexed and the foot in plantar flexion, the graft was secured using a Bunnell suture with removable steel wire, followed by additional reinforcement with a figure-of-eight suture [76].

Stage two consists of soft tissue reconstruction performed using a posterior tibial artery perforator flap [77]. The flap size is tailored to the wound with the pivot point located at the relevant perforator along the line connecting the medial tibial condyle, medial malleolus, and Achilles tendon [78]. The flap is elevated to the deep fascia while preserving the perforators, rotated to cover the defect, and sutured in place. The donor site was resurfaced using a split-thickness skin graft.

### 5.5. Postoperative Care

Postoperative care included immobilization in plantar and knee flexion for six weeks, removal of the internal wire thereafter, and progressive rehabilitation. A full return to normal activity is typically achieved within 6 to 12 months. After approximately six weeks, all patients, regardless of age, should commence physiotherapy.

### 5.6. Efficacy of Achilles Tendon Reconstruction Using the ATL Technique with Vascularized Fascia Lata

The application of vascularized tissues in tendon reconstruction offers clear advantages over the use of local flaps combined with non-vascularized fascial grafts. Vascularized grafts are believed to enhance wound healing, improve graft viability, support rapid tendon integration, and reduce the risk of postoperative infection [70,79]. Composite free flaps, most notably, the anterolateral thigh (ALT) flap incorporating vascularized FL, enable simultaneous reconstruction of both the Achilles tendon and the surrounding soft-tissue deficit within a single operative session, often facilitated by a two-team surgical approach [70,80]. The efficacy of this technique is further supported by postoperative (MRI) magnetic resonance imaging, which typically demonstrates satisfactory tendon regeneration, characterized by normal signal intensity and sharply defined tendon margins in axial and sagittal sequences without evidence of peritendinous fibrosis [68]. Although some authors advocate adjunctive tendon transfers to reinforce the reconstruction, such supplementary procedures may be unnecessary when a vascularized fascial graft is employed, thereby preserving the local tendon structures. The single-stage reconstructive strategy can reduce the operative time and overall costs, achieve a durable functional outcome, allow return to routine activities, minimize donor-site morbidity, and produce an acceptable aesthetic appearance without requiring subsequent contouring procedures [68,81]. Microsurgical composite free-flap reconstruction, such as the ALT flap with vascularized FL, represents a reliable and effective option for complex cases involving combined Achilles tendon loss and soft tissue deficiency. Reconstructions utilizing FL have shown high success rates, contribute to improved stability of the ankle joint, and may reduce the likelihood of future tendon-related complications [82].

## 6. The Potential of FL in the Reconstruction of Joints, Tendons, and Ligaments of the Lower Limb

Although numerous surgical procedures have demonstrated that the FL is suitable for knee tendon reconstruction, the number of reported clinical cases and variations in its application remain relatively limited. Therefore, this subsection focuses on the documented uses of FL, highlighting its versatility and considerable reconstructive potential, which may yet be fully explored.

### 6.1. Reconstruction of Patellar Tendon Using ATL Flap with FL

Single-stage reconstruction was performed using an ALT myocutaneous flap with vascularized FL graft. After Doppler-guided perforator identification, a composite ALT flap was harvested and the FL was rolled and secured to reconstruct the patellar tendon. Each ALT flap must be prepared with meticulous care, preceded by precise limb measurements to ensure proper planning [83]. The vastus lateralis covered the joint, the skin paddle closed the soft tissue defect, and the pedicle was anastomosed to the anterior tibial artery. A composite anterolateral thigh (ALT) myocutaneous flap incorporating vascularized Fl can be successfully employed for single-stage reconstruction. This combined flap offers a resilient skin component, robust perfusion of the vastus lateralis muscle, and well-vascularized fascial layer. The skin paddle of the ALT flap allows reliable soft-tissue coverage without the need for additional skin grafting [80]. Moreover, the vascularized FL contributed sufficient tensile integrity, and its enhanced blood supply compensated for the deficient patellar tendon [84]. For myocutaneous grafts, not only is VL combined with FL commonly used, but there are also reported cases in which TFL has been utilized together with FL [85].

### 6.2. Reconstruction of ACL Using Combined Graft of Hamstring and FL

In this procedure, the FL is employed for lateral extra-articular tenodesis (LET). Biomechanical studies have demonstrated that LET effectively restores normal knee kinematics and substantially reduces residual anterolateral rotational instability in ACL-reconstructed knees when combined with an anterolateral reconstruction [12]. The patient was positioned supine under anesthesia with the knee flexed at 90°, and a proximal tourniquet was applied. Hamstring tendons were harvested, doubled or tripled, and sutured, while an FL strip was obtained for LET; femoral and tibial tunnels were drilled in an outside-in fashion, and both grafts were routed through the tunnels with the FL passing deep to the lateral collateral ligament. Fixation was achieved with interference screws: femoral in full extension and tibial at 30° flexion, followed by arthroscopic confirmation of graft placement and stability [86]. Several studies have reported that allogeneic FL can be used as an alternative to autologous hamstring grafts for ACL reconstruction, with the proximal ends secured using an endobutton with three-strand sutures and the distal ends attached to an artificial ligament. However, patients undergoing ACL reconstruction with more harvested hamstring tendons demonstrate greater loss of active knee flexion and a shift in peak torque to a more extended angle [87].

The FL is well suited for soft-tissue grafting because of its favorable anatomical location and reliable vascularity. Several reports have described the successful use of FL in managing anterior tibial muscle ulcerations, where autologous FL patch grafting promotes healing and restores soft-tissue continuity [88,89,90]. Owing to its high tensile strength, the FL also provides excellent structural support, enabling effective reconstruction of the heel or dorsal foot regions [90,91].

Moreover, the ALT flap combined with the FL, previously discussed in the context of Achilles tendon reconstruction, is equally valuable for addressing soft-tissue defects in poorly vascularized areas of the lower limb [92].

## 7. TFL Muscle: A Valuable Asset in Reconstructive Surgery

The TFL is a clinically valuable muscle that plays a critical role in stabilizing the hip and knee during gait and stance. It contributes to balancing body weight, supports the non-weight-bearing limb, and assists in hip abduction, flexion, and internal rotation [19,25]. Owing to its morphology and anatomical location, TFL is well suited for various surgical applications, including tissue reconstruction and aesthetic enhancement.

### 7.1. TFL Muscle-Pedicle Bone Grafting

TFL muscle-pedicle bone grafting offers substantial advantages for hips in Ficat–Arlet Stages II and III. The rationale for this procedure is based on three key principles. Decompression of the femoral head relieves the increased intraosseous pressure, which functions similar to compartment syndrome, thereby restoring circulation compromised in the pathogenesis of the disease [93]. Excision of necrotic tissue by removing the devitalized bone impedes revascularization of the femoral head. Biological reconstruction of the defect involves filling the post-core decompression cavity with a vascularized TFL muscle pedicle and iliac crest segment, which serves as a viable osteoinductive cancellous graft. This graft provides mechanical support to the subchondral surface, while enhancing the revascularization process [94].

### 7.2. Femoral Head Necrosis

Osteonecrosis of the hip, also known as avascular necrosis, is a relatively common condition, especially among young and middle-aged adults. It is a significant cause of hip pain and disability, and often leads to the need for hip replacement surgery. The risk of osteonecrosis is significantly higher in early adulthood and middle age than in pediatric patients, in whom avascular necrosis typically demonstrates spontaneous resolution [95] (Table 2).

Considering the potential complications associated with hip replacement procedures, including infection, thromboembolic events, dislocation, and implant failure, there is growing interest in exploring alternative treatment modalities that may offer improved outcomes for patients. Despite various treatment modalities, approximately 5–12% of patients with avascular necrosis (AVN) ultimately require total hip arthroplasty [99]. In early stage AVN, head-preserving interventions are recommended because of their ability to decrease intraosseous pressure and improve femoral head perfusion [100,101].

### 7.3. Understanding Avascular Necrosis Through the Ficat–Arlet Scale

Navin Kumar Yadavs and Baksi et al. studies conducted in India during the 1991, 2009, and 2024 in locations including Calcutta, Gorakhpur, and Kolkata report on patients undergoing avascular necrosis (AVN) treatment using a bone graft with an attached TFL flap [101,102,103]. Across these studies, 253 patients were treated, involving 283 femoral heads affected by necrotic changes. The average age of the patients ranged from 32 to 35 years, with the youngest being 12 years and the oldest being 60 years. The severity of AVN ranged from Stage I to IV according to the Ficat–Arlet classification (Table 3). Patients with Stage IV disease underwent standard total hip arthroplasty (THA).

The primary indications for surgery are pain and discomfort localized to the hip and groin, often radiating to the knee [100]. Symptoms are aggravated by walking and sitting cross-legged, and in some cases, they are present even at rest. The range of motion of the affected hip is notably limited, particularly in more advanced stages of osteonecrosis [105]. Although, 70–90 degrees of flexion is often retained, other movements are significantly restricted or absent. The preservation of at least one direction of movement, especially flexion, is considered critical for the feasibility of femoral head-preserving procedures [101]. The average duration of symptoms prior to surgery ranges from 17.5 to 18.8 months [102].

### 7.4. Multimodal Diagnosis Using X-Ray, CT, MRI in Avascular Necrosis

Osteonecrosis is diagnosed based on clinical appraisal and radiological investigations [101,102,103]. Given the potential for bilateral involvement seen in 50% of cases of idiopathic bone necrosis and in as many as 80% of steroid-associated necrosis cases, a thorough patient history is considered essential in raising clinical suspicion and guiding further diagnostic evaluation [106]. Imaging assessment of osteonecrosis should initially include plain radiography, given its low cost and widespread availability.

### 7.5. Type of Imaging Best for AVN Detection

There is ongoing debate regarding the most appropriate imaging modality for the evaluation of osteonecrosis. Although differing viewpoints persist, MRI is widely regarded as the gold standard, offering superior sensitivity for detecting early pathological changes and accurately delineating areas of necrotic bone [107]. Nevertheless, conventional MRI has been reported to produce false-negative results during the early stages of necrosis. During this period, mummified bone marrow may exhibit no appreciable signal alterations and early marrow necrosis can still present with fat-like hyperintensity, potentially masking the initial pathological changes [108]. Although both X-ray and Computed Tomography (CT) have lower sensitivity than MRI and typically detect necrotic changes only in more advanced stages, radiographic findings are often sufficient to obviate the need for further imaging [109].

Very encouraging preliminary results have been demonstrated with dynamic gadolinium-enhanced subtraction (DGS) MRI, allowing the depiction of bone perfusion with increased temporal, spatial, and contrast resolution [110]. Scintigraphy is useful when there is no easy access to MRI [111].

### 7.6. Radiologic Features Distinguishing Normal Anatomy from Disease

In osteonecrosis, radiographic abnormalities are typically evident at presentation. Owing to the lack of normal resorption, necrotic bone appears sclerotic in comparison with adjacent structures. In contrast, the acetabulum and femoral metaphysis demonstrate relative osteopenia, a finding attributed to both disuse and the localized hypervascular response characteristic of the disorder. As the condition progresses, a subchondral radiolucent zone and early anterior femoral head collapse become apparent, with these changes most effectively depicted in the frog-leg lateral view [112]. The diagnosis was confirmed by histopathological examination of the subarticular bone obtained from the femoral head during surgery in all cases. Clinical evaluation pre- and postoperatively was performed using the hip rating system of the Hospital for Special Surgery (HSS), which measures pain, walking, muscle power, and range of motion and function. Each function was scored from 0 (worst) to 10 (normal) [101,102,103,109]. The HSS hip rating system is a clinical tool used to evaluate the outcomes of hip surgery, particularly total hip arthroplasty. It is widely used in orthopedic research and clinical practice to assess patient function, pain, and overall surgical success [113].

### 7.7. Surgical Procedure

**Figure 4:**
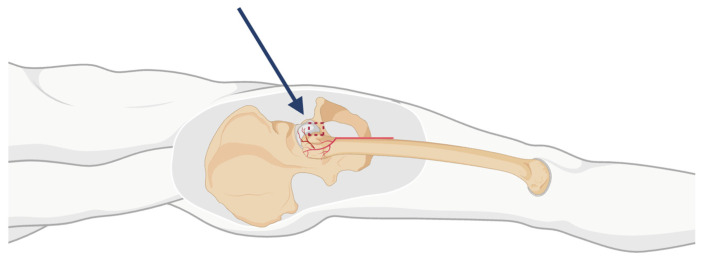
Anterolateral approach used to preserve arteries and vessels (patient in supine position).

**Figure 5:**
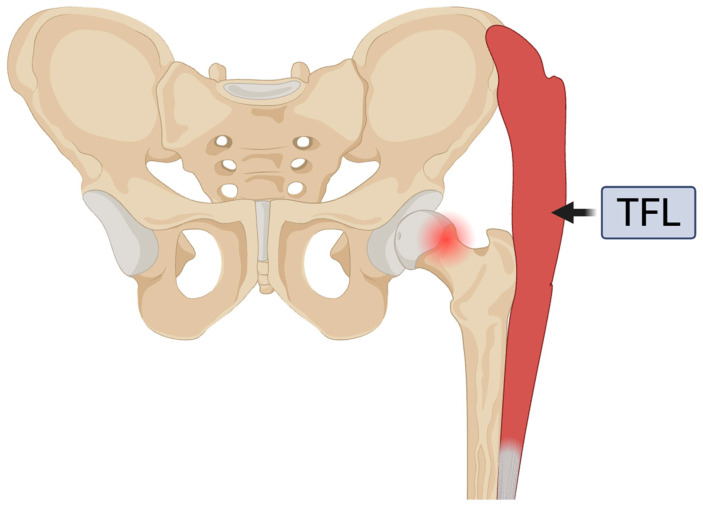
Region of the femoral head affected by AVL. Diagram illustrating the anatomical course of the muscle used for grafting.

The procedure involves the exposure of the anterolateral aspect of the proximal femur near the hip joint. This approach is essential for adequate visualization of the femoral neck and head. The anterolateral incision was also chosen to minimize disruption of the posterior retinacular vessels, thereby preserving the vascular supply to the femoral head [114] (Figure 4), which are small but critical vessels that supply blood to the femoral head, particularly within the hip joint. They originate from the deep femoral artery and its branches, the medial and lateral femoral circumflex arteries, traverse the retinacula, and fibrous extensions of the joint capsule reach the femoral head [115]. Intraoperative injury to these vessels may compromise perfusion, increasing the risk of avascular necrosis (AVN), graft failure, or delayed recovery. Additionally, this approach provides access to the anterolateral quadrant of the femoral head, which is the most commonly affected [101] (Figure 5).

**Figure 6:**
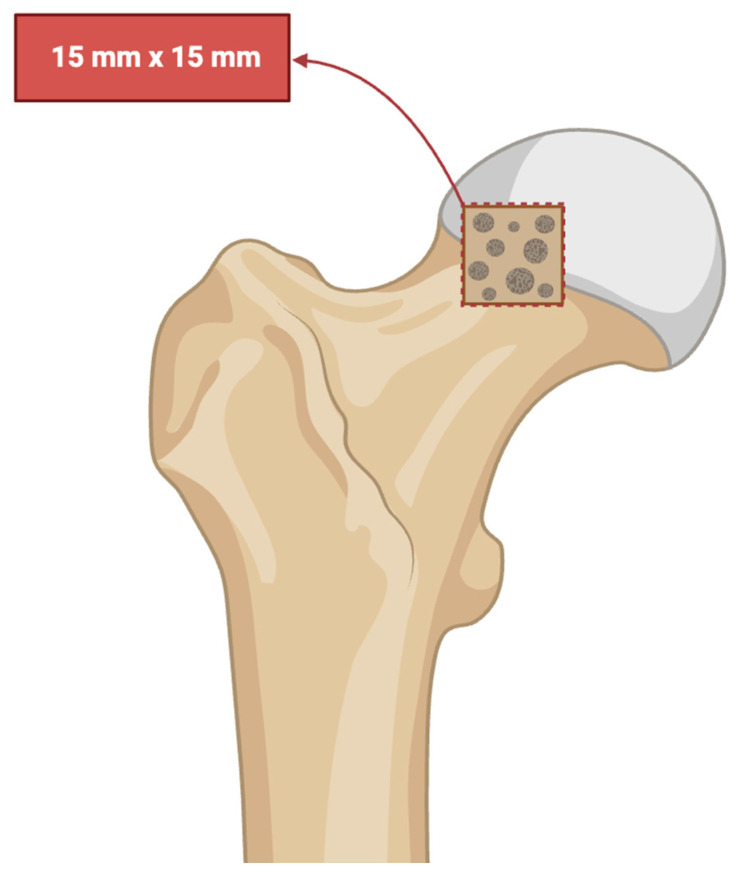
Holes made to enhance vascularization.

To access the necrotic region for removal, a 15 mm ×15 mm bone window was created near the head-neck junction. In cases of idiopathic necrosis, the synovium, which is typically edematous and congested, is excised. Marginal osteophytes are resected from the femoral head, and in advanced stages, cheilectomy is performed on the superolateral aspect of the femoral head, medial to the acetabular rim. This intervention aims to enhance the range of hip abduction and rotation [116]. The articular surface of the femoral head was then inspected for alterations in color and contour as well as for evidence of erosion or softening. Multiple drill holes were made through the window to enhance revascularization [117] (Figure 6). The skin incision was then extended to the middle third of the iliac crest, allowing for careful dissection and downward rotation of a 20 × 25 mm bone segment containing the TFL muscle-pedicle [102].

**Figure 7:**
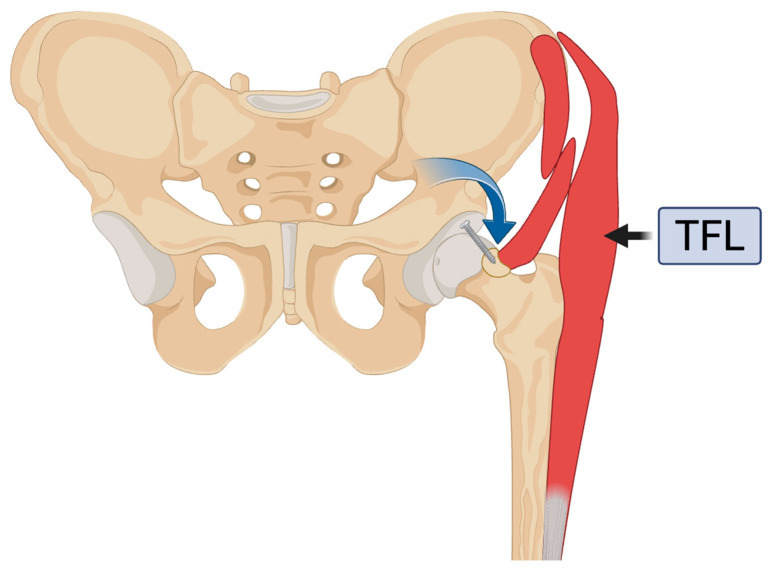
Shaped graft fitted to the window, secured in place by screw.

The prepared muscle-pedicle bone graft receives its blood supply from the superior gluteal artery and the ascending branch of the lateral circumflex femoral artery [118]. Care was taken to avoid overstretching the vascular pedicle. After shaping the bone segment to fit the window, it was secured in place using a 4 mm cannulated screw (Figure 7). The joint capsule was repaired, and the wound was meticulously closed in layers with a suction drain left in place [101].

### 7.8. Postoperative Treatment

Patients were allowed to sit upright in bed for 24 h postoperatively. The first dressing change and drain removal were performed within 48 h. The operated hip is maintained at 30° of flexion and neutral rotation, a position recommended for approximately one week to reduce tension on the vascular pedicle [119]. Across studies, isometric exercises for the quadriceps and gluteal muscles are initiated on the first postoperative day once pain is under control [101,102,103]. The sutures were removed on the 10th postoperative day. Range-of-motion (ROM) exercises are typically initiated two weeks after surgery [120]. During the first six weeks postoperatively, toe touch and non-weight-bearing mobilization were permitted. From weeks 6 to 12, the patient progressed to partial weight-bearing with crutches. Full, unsupported weight-bearing is generally allowed between 15 and 24 weeks postoperatively; however, there is variability in the literature. Some authors recommend delaying full weight-bearing until 5 to 6 months post-surgery, particularly in patients with corticosteroid-induced osteonecrosis, for whom an even longer delay may be warranted [102].

### 7.9. Follow-Up Interval

Follow-up evaluations were routinely performed at 6, 12, and 24 weeks, as well as at one year postoperative. In some cases, however, the final follow-up was extended to 10–12 years with an average duration of 86 months. Patient outcomes were assessed using the Harris Hip Score (HHS), and radiographic imaging was reviewed for evidence of osteoarthritic progression [121].

### 7.10. Improvement and Healing After TFL Flap

In studies by Baksi et al. (1983–2009) [102,103,120], radiological improvement was evaluated based on the following parameters: reduction in the density of the necrotic segment of the femoral head, normalization of previously rarefied areas, restoration of the physiological trabecular architecture, resolution of the crescent sign, healing of cystic lesions and intraosseous fracture lines within or adjacent to the necrotic zone, improved femoral head morphology, particularly following cheilectomy, and preservation or enhancement of the radiographic joint space.

Clinical and outcomes were mostly classified using the HHS:

Excellent: Absence of hip pain, minimal or no functional limitations, hip score of 90–100 points, and radiologically normal femoral head improved morphology [122].

Good: No hip pain, normal or mildly impaired gait, some limitation in range of motion or function, and hip score of 80–90 points. Mild femoral head flattening or early, asymptomatic degenerative changes may be present [123].

Fair: Intermittent mild hip pain, particularly during ambulation, moderate restriction in mobility, hip score of 70–80 points; limited radiological evidence of healing, and signs of mild to moderate femoral head flattening and joint degeneration [124].

Poor: Constant hip pain, ankylosis of the joint, hip score < 70, absence of radiological healing, noticeable flattening of the femoral head, and moderate to advanced degenerative joint changes [122].

Analysis of patient outcomes in these studies [101,102,103] demonstrated a high success rate, with excellent or good results observed in 85.4% of cases. Only a small proportion of the patients (14.9%) experienced fair or poor outcomes (Table 4 and Table 5).

### 7.11. Efficiency of TFL Compared to Alternative Muscles in Femoral Head AVN

Vascularized fibular grafting has gained popularity as a head-preserving option for osteonecrosis, offering improved vascular supply to the femoral head. However, this approach is technically demanding, requires specialized training, and has a steep learning curve. Reported failure rates range from 5% to 30%, depending on surgical expertise and case selection [101]. In recent years, the use of non-vascularized bone grafts such as tibial or fibular autografts and allografts has declined significantly owing to their high failure rates. In a study by Keizer et al. (2006) [125], tibial autografts and fibular allografts were used to treat femoral head osteonecrosis in 78 patients. Clinical failure was observed in 54% of the cases, underscoring the limitations of non-vascularized techniques [125]. On the other hand, vascularized fibular grafting, while effective, presents several potential disadvantages. These include longer operative time, requirement for microsurgical expertise, more extensive surgical scar, and higher donor site morbidity. Reported complications include ankle instability, toe clawing, subtrochanteric fractures, and heterotopic ossification [94].

In contrast, TFL-pedicle bone grafting combined with iliac crest segment offers a technically straightforward alternative that promotes early revascularization. The TFL graft is generally preferred because of its superior vascular supply, especially when the necrotic area is extensive [126]. In cases where necrosis affects the posterior aspect of the femoral head, either a quadratus femoris or gluteus medius muscle-pedicle graft is used [127]. TFL muscle-pedicle bone grafting has emerged as a promising and technically accessible alternative, particularly for patients with Ficat–Arlet stages II and III. This technique utilizes a portion of the iliac crest and does not require microsurgical expertise or specialized equipment, yet provides the benefit of enhanced vascularity akin to vascularized bone grafts [94,101]. Baksi et al. demonstrated favorable outcomes using muscle pedicle grafts derived from the quadratus femoris, gluteus medius, sartorius, and TFL in various stages of femoral head osteonecrosis [120]. Among these, the TFL graft showed superior biomechanical support and revascularization potential in the pre-collapse stages, effectively preventing femoral head collapse [102]. Core decompression plays a dual role in this regard. It not only provides histological insight into early ischemic changes but also alleviates symptoms in the pre-collapse stage by reducing intraosseous pressure. Except in rare cases, conservative management has a limited role in femoral head osteonecrosis, and surgical intervention is generally considered inevitable [128]. TFL muscle-pedicle bone grafting not only improves vascularity but also offers mechanical reinforcement to the subchondral bone, aiding in structural preservation. The procedure is less technically demanding than free vascularized grafting, making it more accessible to general orthopedic surgeons while offering high success rates [129]. Clinical and radiological outcomes have consistently demonstrated that TFL muscle-pedicle bone grafting is an effective joint-preserving option, particularly in younger patients for whom total hip arthroplasty may not be ideal. The procedure yields significant pain relief, functional improvement, and disease stabilization in both the early and selected advanced stages of osteonecrosis, provided there is no significant arthritic involvement [101,102,103].

## 8. Discussion

Both TFL and FL have emerged as valuable autologous materials for contemporary reconstructive surgeries. Recent studies have highlighted their excellent mechanical strength, biological viability, and adaptability, underscoring their suitability for a variety of demanding procedures [25,42]. As a muscular structure with a reliable vascular anatomy, the TFL has long been used in innovative soft-tissue reconstruction. Its predictable pedicle, favorable location, and robust perfusion make it an established option for the management of abdominal and thoracic wall defects, chronic ulcerations, and complex soft-tissue infections [130,131]. TFL is therefore widely recognized and well understood by reconstructive surgeons, with a substantial body of literature demonstrating its versatility and clinical reliability [132]. However, this review emphasizes the role of the TFL and FL in lower-limb salvage, where both structures contribute not only to local biomechanics but also to weight transmission and postural stability. Their anatomical functions naturally translate into excellent reconstruction capabilities. A noteworthy example is the application of TFL grafts in the treatment of avascular necrosis (AVN) of the femoral head. The clinical outcomes have been encouraging, with reported success rates surpassing those of other muscular pedicle grafts. For instance, the gluteus minimus pedicled graft demonstrated approximately 60% symptomatic improvement in Ficat–Arlet stages II–III, whereas TFL-based reconstructions achieved success rates exceeding 85% in comparable cohorts [133].

Similarly, Zhang et al. (2019) presented a series of 106 hips treated with a sartorius muscle-pedicle graft, achieving excellent clinical recovery on the HHS (mean 89.18 ± 3.16 at 24 months) [134]. The outcomes were comparable to those obtained with deep circumflex iliac artery bone flaps. Although bone flaps had slightly better outcomes, the authors emphasized the advantages of muscle-based procedures, including shorter operative times, reduced bleeding, and suitability for primary-level centers [134]. Muscle-based grafts are beneficial when joint stability, mechanical support, and enhanced vascularity are required. However, not all cases require invasive approaches. Treatment selection must account for patient-specific factors such as age, general health, and disease extent. Alternatives, such as core decompression (CD) and its evolving variants, have been widely practiced [135]. Although techniques such as the trapdoor method allow direct visualization of the femoral head, their invasiveness and the risk of compromising femoral neck vascularity limit routine use. Nevertheless, ongoing refinements continue to improve their safety profiles [136,137]. The combined use of CD and bone marrow-derived stem cells has further improved outcomes by enhancing biological regeneration [138]. Other alternatives include vascularized and non-vascularized bone grafts, which provide structural support and promote osteogenesis, although non-vascularized cortical strut grafts have fallen out of favor [139]. The selection of TFL- and FL-based flaps depends largely on clinical circumstances and desired functional outcomes. Despite their advantages, these tissues are not universally selected, particularly in procedures such as anterior cruciate ligament (ACL) reconstruction, where the quadriceps tendon or hamstring grafts remain a common choice [140,141]. Nonetheless, the mechanical resilience and favorable handling characteristics of FL make it a highly effective material when appropriately indicated. The Achilles tendon is another area where the FL has proven to be valuable. Its tensile strength and resistance to overload make it an excellent option for reconstructing large tendon defects, while simultaneously enhancing ankle stability. Although several local tendons such as the plantaris, flexor hallucis longus, peroneus brevis, and flexor digitorum longus are frequently used in tendon transfer [142,143], defects exceeding 6–10 cm often require more robust solutions, such as free tendon grafts, fascia lata plasties, allografts, and synthetic substitutes [144]. Although effective, muscle flaps sometimes require sacrificing major arterial branches and therefore carry a risk of donor-site ischemia, as demonstrated with dorsalis pedis flap reconstruction [145]. In such situations, FL serves as a biomechanically favorable alternative that avoids significant donor site compromise. The anterolateral thigh (ALT) fasciocutaneous flap remains one of the most versatile options for Achilles tendon reconstruction) [62,70,71,73]. It must be emphasized, however, that the clinical evidence supporting these reconstructive applications is limited in both quantity and quality. The reports underpinning the use of the ALT/FL flap in Achilles tendon reconstruction are case reports and small case series describing only a few patients each (for example, one, three, one and six patients in refs. [62,70,71,73], respectively), rather than controlled or comparative studies. Similarly, the outcome and necrosis-stage data summarized in Table 4 and Table 5 derive from a small number of single-center, retrospective case series [101,102,103]. Consequently, these studies are subject to the biases inherent in uncontrolled, single-center designs with limited follow-up and heterogeneous reporting, and no randomized controlled trials are currently available in this field. The favorable results reported should therefore be regarded as preliminary observations that require confirmation in larger, prospective, and ideally comparative studies before firm conclusions can be drawn about the clinical superiority or reliability of FL- and TFL-based reconstruction. A key advantage is the ability to simultaneously harvest a segment of the FL to create a durable tendon substitute. To minimize donor-site morbidity, fascia from the vastus lateralis or rectus femoris may be used as an alternative [67,81]. Although FL is not used as frequently as TFL in some reconstructive domains, its documented applications, particularly in lower extremity reconstruction, highlight its considerable and often underappreciated utility. As a structurally robust, collagen-dense tissue with favorable biological characteristics, the FL represents an exceptionally promising graft for both soft tissue and osteotendinous reconstruction in modern limb surgery.

## 9. Conclusions

The FL and its related components form an essential system for the lateral thigh. Its mechanical durability, provided by its strong collagen structure and rich vascularization, makes it a versatile grafting material for reconstructive surgery. Although there are a few high-quality clinical studies that endorse the evidence that FL is an immensely valuable asset in contemporary orthopedic and reconstructive surgery, it is largely underutilized, yet highly promising material. Further prospective studies are required to optimize surgical protocols and expand their clinical potential. Future research should focus on refining surgical techniques, improving graft integration, and exploring innovative regenerative strategies to boost the effectiveness of the current methods.

## 10. Limitations

Research on FL and its components is limited by several important factors. The ability to generalize anatomical characteristics to a broader population is restricted by only a few available studies that rely on small patient groups, which may not fully represent the different patterns of vascularization and innervation observed in living patients. The amount of cadaveric material used was insufficient. Additionally, long-term clinical data are lacking, and although the initial results are promising, durability, complication rates, and functional outcomes remain inadequately documented. Biomechanical analyses of not only the FL but also of the ITB and TFL remain incomplete, so it is not yet possible to predict its integration with host tissue and regeneration with certainty. Because of its unique biological structure, FL is a promising, yet underutilized material. Therefore, more long-term studies are needed to increase the effectiveness of modern reconstructive surgery.

## Figures and Tables

**Table 1:** The fascia lata complex function summary.

Element	Biomechanical Aspect
The FL	load transfer, stability and joint connections
The TFL	hip flexion, abduction and rotation, knee stability and flexion beyond 30°
The ITB	hip lateral stability, knee anterolateral stability, knee flexion and extension, pivot shift control
The GMAX	pelvis lateral stability, hip extension and rotation, loads transfer, sacroiliac joint stability
The retinacula	knee stability, patella movement control

**Table 2:** Etiological factors and mechanisms involved in the development of osteonecrosis.

Cause Type	Examples and Mechanisms
traumatic	Femoral neck fractures, hip dislocations, or iatrogenic injury can disrupt the medial femoral circumflex artery, causing vessel rupture, thrombosis, or increased intracapsular pressure leading to ischemia [96].
non-traumatic corticosteroid use	Long-term or high-dose therapy alters lipid metabolism, promotes fat emboli, increases intraosseous pressure, and exerts toxic effects on bone cells and endothelium, reducing perfusion. Common in SLE, nephrotic syndrome, and post-transplant patients [97].
non-traumatic alcohol abuse	Chronic excessive intake causes fatty marrow infiltration, fat embolism, increased intraosseous pressure, and direct osteocyte toxicity, impairing bone repair [98].
non-traumatic medical conditions	Includes HIV, autoimmune diseases, chronic kidney disease, post-transplant states, sickle cell disease, Gaucher disease, hyperlipidemia, and coagulopathies. Mechanisms involve vascular occlusion, endothelial injury, and altered bone turnover [96].

**Table 3:** Classification FICAT–ARLET and its stages for femoral head necrosis [104].

Modality/Feature	Stage 0	Stage I	Stage II	Stage III	Stage IV
plain radiograph	normal	normal or minor osteopenia	mixed osteopenia and/or sclerosis and/or subchondral cysts, without any subchondral lucency	crescent sign and eventual cortical collapse	end-stage with evidence of secondary degenerative change
MRI	normal	edema	geographic defect	crescent sign and eventual cortical collapse	end-stage with evidence of secondary degenerative change
bone scan	normal	increased uptake	increased uptake	increased uptake	
clinical symptoms	none	pain in the groin	pain and stiffness	pain and stiffness	pain and limp

**Table 4:** Clinical outcomes reported across the analyzed case series. Values are aggregated from individual case series [101,102,103].

Results	No. of Cases	%	95% Cl for *n* = 283
Excellent	135	48	41.88–53.52%
Good	106	37.4	31.82–43.10%
Fair	35	12.4	8.53–16.20%
Poor	7	2.5	0.66–4.28%
All	283	100	---

**Table 5:** Distribution of necrosis stages across the analyzed case series. Values are aggregated from individual case series [101,102,103].

Stage of Necrosis	No. of Cases	%	95% Cl for *n* = 283
I	5	2	0.76–4.07%
II	93	32.9	27.65–38.53%
III	152	53.7	47.89–59.43%
IV	19	7	4.34–10.25%
V	11	4	2.18–6.82%
VI	3	1	0.36–3.07%
All	283	100	---

## Data Availability

No new data were created or analyzed in this study. Data sharing is not applicable to this article.
